# Comparative methods for PET image segmentation in pharyngolaryngeal squamous cell carcinoma

**DOI:** 10.1007/s00259-011-2053-0

**Published:** 2012-01-31

**Authors:** Habib Zaidi, Mehrsima Abdoli, Carolina Llina Fuentes, Issam M. El Naqa

**Affiliations:** 1 Division of Nuclear Medicine and Molecular Imaging, Geneva University Hospital, CH-1211 Geneva, Switzerland; 2 Geneva Neuroscience Center, Geneva University, CH-1205 Geneva, Switzerland; 3 Department of Nuclear Medicine and Molecular Imaging, University Medical Center Groningen, University of Groningen, 9700 RB Groningen, The Netherlands; 4 Department of Medical Physics, McGill University, H3G 1A4 Montreal, Canada

**Keywords:** PET, Segmentation, Biological tumour volume, Head and neck, Treatment planning

## Abstract

**Purpose:**

Several methods have been proposed for the segmentation of ^18^F-FDG uptake in PET. In this study, we assessed the performance of four categories of ^18^F-FDG PET image segmentation techniques in pharyngolaryngeal squamous cell carcinoma using clinical studies where the surgical specimen served as the benchmark.

**Methods:**

Nine PET image segmentation techniques were compared including: five thresholding methods; the level set technique (active contour); the stochastic expectation-maximization approach; fuzzy clustering-based segmentation (FCM); and a variant of FCM, the spatial wavelet-based algorithm (FCM-SW) which incorporates spatial information during the segmentation process, thus allowing the handling of uptake in heterogeneous lesions. These algorithms were evaluated using clinical studies in which the segmentation results were compared to the 3-D biological tumour volume (BTV) defined by histology in PET images of seven patients with T3–T4 laryngeal squamous cell carcinoma who underwent a total laryngectomy. The macroscopic tumour specimens were collected “en bloc”, frozen and cut into 1.7- to 2-mm thick slices, then digitized for use as reference.

**Results:**

The clinical results suggested that four of the thresholding methods and expectation-maximization overestimated the average tumour volume, while a contrast-oriented thresholding method, the level set technique and the FCM-SW algorithm underestimated it, with the FCM-SW algorithm providing relatively the highest accuracy in terms of volume determination (−5.9 ± 11.9%) and overlap index. The mean overlap index varied between 0.27 and 0.54 for the different image segmentation techniques. The FCM-SW segmentation technique showed the best compromise in terms of 3-D overlap index and statistical analysis results with values of 0.54 (0.26–0.72) for the overlap index.

**Conclusion:**

The BTVs delineated using the FCM-SW segmentation technique were seemingly the most accurate and approximated closely the 3-D BTVs defined using the surgical specimens. Adaptive thresholding techniques need to be calibrated for each PET scanner and acquisition/processing protocol, and should not be used without optimization.

## Introduction

Image-guided adaptive radiation therapy using modern technology has emerged as a promising approach to dose escalation in pharyngolaryngeal squamous cell carcinoma [1]. For many years, anatomical imaging was used to delineate gross tumour volumes for radiotherapy treatment planning. However, the wide adoption of PET/CT in the clinical setting and the efficacy of ^18^F-FDG PET imaging in a wide variety of malignant tumours with high sensitivity, specificity and accuracy stimulated the use of this technology in radiation therapy [2]. PET/CT has been proven to provide superior sensitivity compared to CT simulation, which in some cases might miss some regions that appear suspicious on PET images including the detection of distant metastases, and to shed light on the actual tumour volume, which might in reality be smaller or bigger on the PET study than on the CT scan alone. In addition, in many studies the findings of anatomical and molecular imaging modalities have disagreed, and the addition of PET has been shown to result in modification of the treatment plans in a substantial number of clinical studies [3]. Another important consideration is the reduction in inter- and intraobserver variability achieved with PET for delineation of the biological tumour volume (BTV) compared to anatomical imaging techniques [4].

Accurate target volume delineation using PET has proved to be a challenging task owing to the intrinsic properties of PET data. As a result, a wide variety of image segmentation techniques have been proposed [5]. However, validation of accuracy (fidelity to the truth) and precision (reproducibility) of these algorithms still remain unresolved and require further research and development effort. A rather challenging and even problematic issue for validation of segmentation algorithms is the identification of a gold standard (i.e. the benchmark). The lack of guidelines established by nuclear medicine and radiation oncology professional societies renders this task more complex. Four approaches have been used in the literature to assess the accuracy of PET image segmentation techniques. These include manual delineation performed by experienced physicians, the use of simulated or experimental phantom studies where the tumour volume and spatial extent are already known, comparison with correlated anatomical gross tumour volumes defined on CT or MRI, and comparison of tumour volumes delineated on clinical PET data with actual tumour volumes measured on the registered macroscopic specimens derived from histology (where a PET scan was undertaken before surgery).

Evaluation and validation of PET image segmentation techniques using simulated or experimental phantom studies is very popular thanks to the availability of a wide variety of static and dynamic physical phantoms and comprehensive anatomical and physiological anthropomorphic models of the human body [6]. The use of the macroscopic surgical specimen for validation of PET image segmentation techniques is one of the most promising approaches reported so far for clinical studies [7, 8] provided deformation related to the shrinkage of the specimen after surgical excision are taken into account [9].

We present here an assessment of the performance of four categories of ^18^F-FDG PET image segmentation techniques for pharyngolaryngeal squamous cell carcinoma in clinical studies, where the 3-D contour defined on the surgical specimen served as the reference.

## Materials and methods

### Segmentation methods

PET image segmentation methods can be divided into four broad categories based on the underlying methodology: (1) thresholding methods, (2) variational approaches, (3) stochastic modelling-based techniques, and (4) learning methods. For this study we selected representative methods from each category. Five thresholding methods, one variational method, one stochastic modelling method and two learning methods were selected. These methods are reviewed below briefly for completeness. Detailed descriptions of these methods can be found in the recent review by Zaidi and El Naqa [5]. PET segmentation algorithms were implemented using MATLAB (Mathworks, Natick, MA) with a Mac Pro 8 quad-core Intel Xeon “Nehalem” processor and 64-bit architecture running a Snow Leopard operating system.

#### Thresholding

This is the simplest and most widely used algorithm for PET image segmentation, in which a threshold value (*T*) is selected to separate the lesion foreground from a noisy background. In these methods, the PET image voxels are usually converted into standardized uptake values (SUVs) prior to threshold selection. SUV is a decay-corrected measurement of activity per unit mass of tissue adjusted for administered activity per unit body weight. Among the earliest thresholding methods used is thresholding by percentage (40–50%) of the maximum intensity based on phantom studies of stationary spheres filled with ^18^F [10]. However, studies have shown that optimal threshold selection is dependent on tumour volume [11, 12]. Therefore, Biehl et al. derived a logarithmic relationship between tumour volume and the optimal threshold using manually delineated CT data in a cohort of lung cancer patients [11]:

1 $$ \% Threshold = {59}{.1 - 18}{.5} \times {\text{log10(tumor volume)}} $$

In order to avoid the controversies associated with fixed thresholds, some authors have suggested adapting the threshold value to the signal-to-background ratio. For instance, Black et al. suggested [13]:

2 $$ Threshold = 0.307 \times ({\text{mean}}\;{\text{target}}\;{\text{SUV}}) + 0.588 $$

Further modifications of Eq. 2 have been proposed to better fit the data. Nestle et al. [14] used the following formula:

3 $$ Threshold = \beta \,{I_{{mean}}} + {I_{{bkg}}}, $$

where *β* = 0.15, *I*_mean_ is the mean intensity of all pixels surrounded by the 70% *I*_max_ isocontour within the tumour and *I*_bkg_ is the mean intensity in a neighbouring organ far from the target and having the highest tracer uptake. More recently, Schaefer et al. [15] proposed the following modification:

4 $$ Threshold = \left( {a\, \times \,SU{V_{{mean(70\% )}}} + b\, \times \,BKG} \right)\,\,/SU{V_{{\max }}}, $$

where *a* and *b* are sphere size and PET scanner-dependent parameters (*a* = *b* = 0.50 for large spheres of diameter ≥3 cm, and as *a* = 0.67 and *b* = 0.60 for small spheres of diameter <3 cm). SUV_mean(70%)_ is the mean SUV of the 70% isocontour and BKG is the background.

#### Variational approaches

These methods attempt to exploit information provided by intensity variation (gradient differences between the foreground lesion and the background) for the segmentation task. Methods based on deformable active contours are receiving more attention in this category for PET image segmentation [16]. As representative of this group, we chose a geometric deformable model approach based on geometric level sets [17] as implemented in the above reference. This approach is based on the geometric concept of evolving level sets by iteratively solving the following evolutionary Euler-Lagrange partial differential equation:

5 $$ \frac{{\partial \phi }}{{\partial t}} = V(\kappa )\left| {\nabla \phi } \right| $$

where *φ* is an implicit function (e.g. a signed distance) that represents the evolving level set, where at the contour $\phi (C) = 0$, *V* is a velocity function proportional to the curvature (*κ*) and inversely proportional to the image gradient. The algorithm starts by some initial contour, then the curve evolves under the influence of the internal (contour curvature) and external (image gradient) forces until it reaches the boundary of the object where balance between the internal and the external forces is achieved.

#### Stochastic modelling

In these approaches statistically significant differences are used to separate uptake in the lesion voxels from that in surrounding normal tissue voxels [18]. The PET segmentation problem in this case is formulated as maximum likelihood estimation of unknown parameters of probability distribution (*f*) of the voxel intensity (can be assumed to be a gaussian distribution) using the expectation-maximization (EM) algorithm and the probability of voxel *x*_*i*_ belonging to class *k*, which is given by:

6 $$ {p_{{ik}}} = \frac{{{\pi_k}{f_k}({{{{x_i}}} \left/ {{{\mu_k},{\sigma_k})}} \right.}}}{{\sum\limits_{{m = 1}}^K {{\pi_m}{f_m}({{{{x_i}}} \left/ {{{\mu_m},{\sigma_m})}} \right.}} }} $$

where *π* are the mixing parameters and *μ* and *σ* are the gaussian mean and standard deviation, respectively. The EM algorithm proceeds in two steps, where the E step is dedicated to the computation of the probabilities and the M step estimates the cluster parameters assuming that the intensity distribution of each class may not be gaussian [19, 20].

#### Learning methods

In this class of methods, the PET segmentation problem is formulated as a learning task to separate uptake in the lesion voxels from that in surrounding normal tissue voxels based on a set of features extracted from the PET image, such as intensity. As a representative method, we chose the unsupervised clustering method based on the fuzzy C means (FCM) algorithm. In this approach, a voxel might belong to more than one class (opposite to classical k-means), and cluster association is built into the algorithm by using fuzzy membership functions [21, 22]. In this scenario, the fuzzy member function at any iteration *n* is given by:

7 $$ u_{{ik}}^{{(n)}} = \frac{{{{\left\| {{{\mathbf{x}}_i} - {\mathbf{c}}_k^{{(n)}}} \right\|}^{{ - 2}}}}}{{\sum\nolimits_{{k = 1}}^K {{{\left\| {{{\mathbf{x}}_i} - {\mathbf{c}}_k^{{(n)}}} \right\|}^{{ - 2}}}} }} $$

and the update for cluster centres is:

8 $$ c_k^{{(n + 1)}} = \frac{{\sum\nolimits_{{i = 1}}^N {{{\left( {u_{{ik}}^{{(n)}}} \right)}^b}{{\text{x}}_i}} }}{{\sum\nolimits_{{i = 1}}^N {{{\left( {u_{{ik}}^{{(n)}}} \right)}^b}} }} $$

where **x**_*i*_ is the feature vector at the *i*th location, ${\mathbf{c}}_k^{{(n)}}$ is the *k*th centroid at the *n*th iteration, and *b* is an exponent >1. A variation of this method was applied by Zaidi et al. [23], in which the algorithm starts with an oversized number of clusters to avoid misidentification of conflicting regions. This is followed by a merging process to reach the desired or natural number of clusters according to a priori anatomical knowledge.

A variation of this approach was successfully used for PET image segmentation [24]. PET images are first smoothed using a nonlinear anisotropic diffusion filter [25] and are then added as a second input to the FCM algorithm to optimize the objective function with knowledge about spatial constraint, thus incorporating spatial information (FCM-S). In addition, a methodology was developed to integrate the *à trous* wavelet transform [26] in the standard FCM algorithm (FCM-SW) to allow handling of’ uptake in heterogeneous lesions. This is achieved by adding a regularization term to the FCM objective function using the transformation result of the PET image by the *à trous* wavelet transform with the aim of incorporating information about lesion heterogeneity.

The influence of the wavelet on voxel clustering update is controlled by *β*_*k*_ coefficients, which depend on two parameters set by the trial-and-error technique. This approach is well established for solving problems where there are multiple chances of obtaining the correct solution. These values were chosen in such a way that the influence of the wavelet filtered image remained most important for most regions of the image showing high tracer uptake, such that there is a stronger influence on the objective function when the current voxel is located inside the tumour [24]. These values were optimized once and were not adjusted again by hand for the particular images being processed.

### Clinical studies

Seven patients with T3–T4 laryngeal squamous cell carcinoma from the Louvain database [7] who had undergone an ^18^F-FDG PET study prior to treatment were included for comparative analysis of the investigated PET image segmentation methods [27]. The patients were immobilized with a tailored thermoplastic mask (Sinmed, Reeuwijk, The Netherlands) attached to a flat table-top to avoid neck motion during scanning. A preinjection transmission scan (10 min) was acquired prior to intravenous injection of 185–370 MBq of ^18^F-FDG, which was followed by a 60-min dynamic 3-D PET emission scan using an ECAT EXACT HR camera (CTI/Siemens, Knoxville, TN). PET data were reconstructed using a 3-D AW-OSEM algorithm (four subsets and eight iterations) following correction for dead time, randoms, scatter, attenuation and radioactive decay.

Patients underwent a total laryngectomy a few days (average 5 days) following the PET study. A special procedure was developed to allow 3-D coregistration of the macroscopic specimens with the imaging modalities. Fresh surgical specimens were placed in a polystyrene cast containing three longitudinally placed wooden rods that were equally spaced in the transverse plane of the specimen and the cast was filled with a 16% gelatin solution and kept at −20°C for 48 h and then at −80°C for at least 72 h. The authors reported that these fixation and freezing procedures did not result in retraction compared to other methods as evident by their animal data [7]. The macroscopic tumour specimens were gathered, frozen and cut into 1.7- to 2-mm thick slices. The 3-D specimen was reconstructed following digitization and realignment of the obtained thin slices also taking into account the material lost during slicing. A semiautomated rigid-body registration algorithm was then used to coregister the PET and macroscopic surgical specimen images [28]. The last step consisted of creating the fully 3-D macroscopic tumour volume by delineating separately on each slice the macroscopic tumour extension. The volume obtained served as the reference for evaluation of the investigated PET segmentation techniques.

**Fig. 1:**
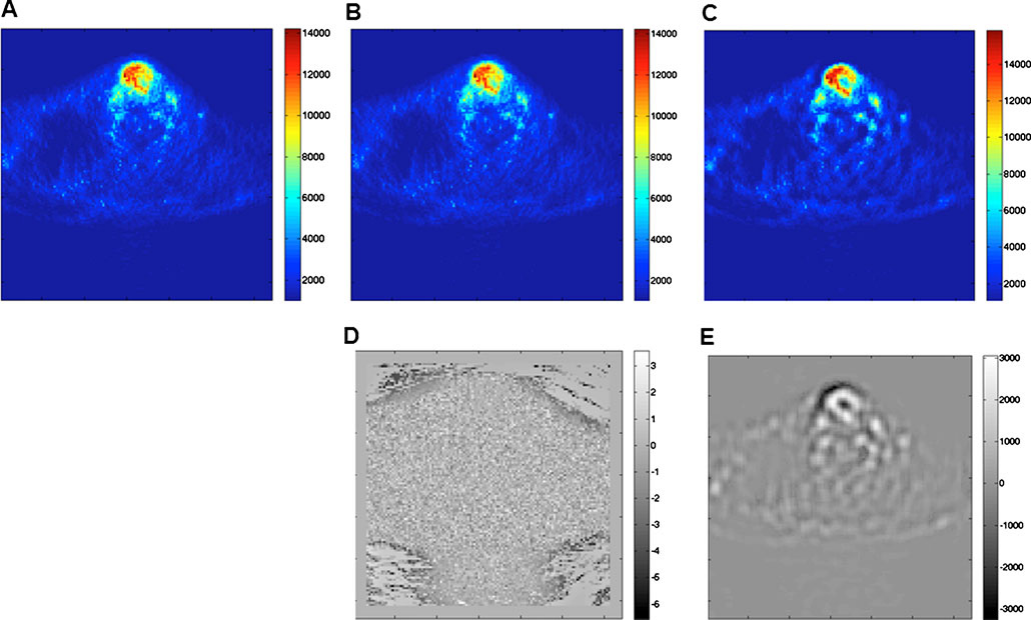
Representative clinical FDG PET images showing the impact of denoising and deblurring steps on visual quality: **a** original image, **b** after denoising, **c** after denoising and deblurring. The difference between the original and processed images after denoising (**d**) and after denoising and deblurring (**e**) are also shown

Following the work of Geets et al. [27], we applied a similar two-step preprocessing scheme to improve the quality of the images for subsequent segmentation. The first step consisted of denoising. This was carried out using a 3-D bilateral filter, which is similar to a gaussian filter but with better edge-preserving properties. Two multiplicative gaussian kernels were used. One kernel acted as a spatially weighting function with full-width at half-maximum (FWHM) 6 mm and the other kernel was adjusted according to local changes in intensity (radiometric differences). Two iterations of subsequent denoising were applied. The second step (deblurring) aimed at compensating for the scanner imperfect point spread function, partial volume effect, and recover lost resolution due to denoising. In this case, a deconvolution process based on the Landweber’s iterative algorithm was applied with a gaussian kernel of FWHM 6 mm and 30 iterations. Parameters that were not clear in the original description given in the above reference were assigned empirically. Figure 1 shows the impact of the denoising and deblurring steps on visual quality of clinical FDG PET images. The intrinsic noise and blur were reduced after performing these operations, resulting in sharper edges and steeper gradients.

### Evaluation metrics

To evaluate the segmentation accuracy of the different methods, we compared volume changes, which is a commonly used method, in addition to the spatial overlap index (SOI), a quantity similar to the Dice similarity coefficient. SOI is defined in terms of the pixel ratio of the overlapping regions, where at any given threshold, SOI values would range from 0, indicating no spatial overlap between two sets of binary segmentation results, to 1 indicating complete overlap [29]:

9 $$ {\text{SOI}} = \frac{{{\text{2(A}} \cap {\text{B)}}}}{{{\text{(A}} + {\text{B)}}}} $$

In addition, classification errors (CE) were computed on a voxel-by-voxel basis for the clinical studies using the following formula [30]:

10 $$ CE = \frac{{\left( {PCE + NCE} \right)}}{{VoIL}}\,\,\, \times \,\,100\% $$

where PCE refers to the positive CEs, including voxels of the background that are classified as belonging to the tumour, and NCE refers to negative CEs, including voxels of the tumour that are classified as belonging to the background, and VoIL is the number of voxels defining the lesion as identified by histology (actual number of voxels reflecting the ground truth). Moreover, the significance of differences between the measured and reference volumes were evaluated using a two-tailed paired *t*-test.

## Results

**Fig. 2:**
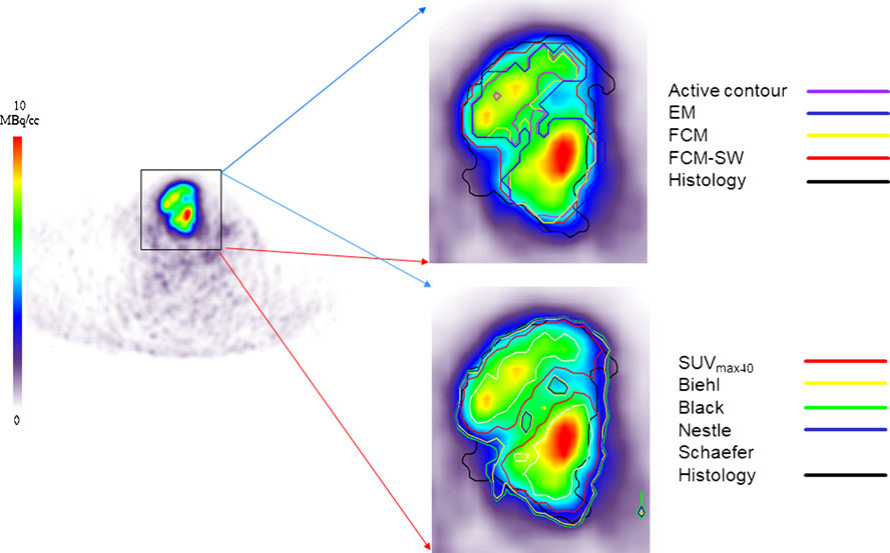
Representative segmentation results for an FDG PET image in a patient presenting with histologically proven laryngeal squamous cell carcinoma showing good boundary estimation. The BTVs defined in the nonhomogeneous lesion with an irregular shape using nine segmentation techniques are shown on the FDG PET transaxial slices. These include five thresholding methods including 40% of SUV_max_ (*SUV*_*max40*_) and the methods of Nestle et al. [14] (*Nestle*), Black et al. [13] (*Black*), Biehl et al. [11] (*Biehl*) and Schaefer et al. [15] (*Schaefer*), the level set technique (*Active contour*), the stochastic EM approach (*EM*), the FCM algorithm (*FCM*), and the FCM-SW variant of the FCM algorithm (*FCM-SW*). The 3-D contour defined on the macroscopic tumour specimen is used as the reference for assessing the performance of the different segmentation techniques

**Fig. 3:**
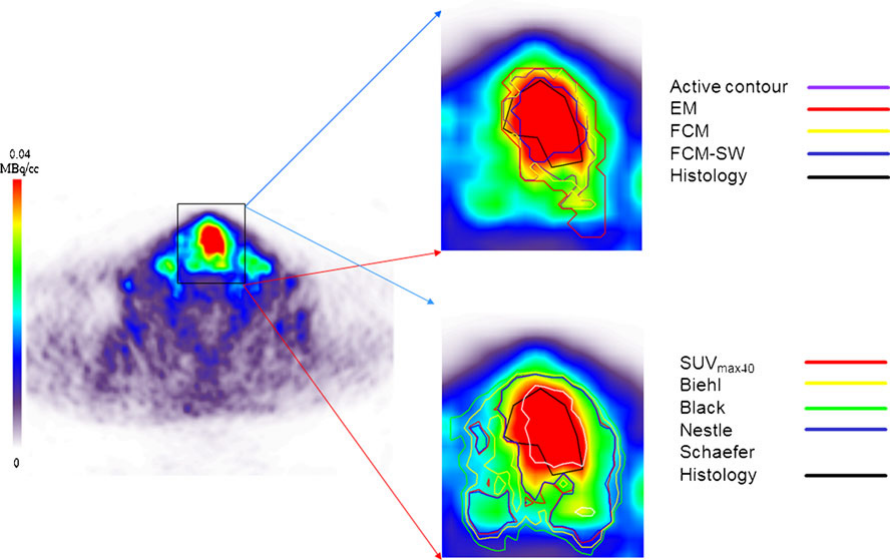
Representative segmentation results for an FDG PET image in a patient presenting with histologically proven laryngeal squamous cell carcinoma to illustrate an extreme case of bad boundary estimation. See Fig. 2 legend for full description of the images

Figure 2 shows example clinical imaging of a pharyngolaryngeal squamous cell carcinoma comparing the target volume definitions obtained using the different segmentation methods with the histology serving as reference. This is a representative case showing good boundary estimation. Automated and semiautomated methods performed well and more accurately reproduced the reference contour defined on histology in the presence of irregular and heterogeneous lesions, whereas large discrepancies were observed with the thresholding techniques. Figure 3 shows another clinical study representative of poor boundary estimation. It should be noted that methods employing tumour size alone [11] or intensity alone [13, 14] were inferior to those utilizing both criteria [15]. Default parameters were applied and fixed across volumes and activity levels to achieve similar references, and no optimization or tuning was carried out in order to represent everyday use. Overall, the FCM-SW algorithm outperformed all techniques despite its moderate performance in the extreme case shown in Fig. 3.

**Fig. 4:**
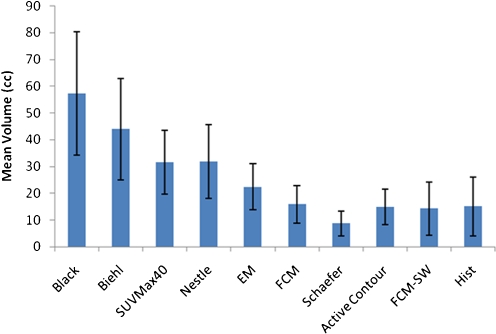
Mean tumour volumes estimated using the different PET image segmentation techniques for the seven clinical cases studied. The mean volume estimated histologically (*Hist*) is also shown. *Error bars* indicate SDs of the means

Figure 4 shows the mean tumour volumes obtained from the surgical specimens together with those obtained using the various PET image segmentation techniques. The mean volume of the surgical specimens was 15.15 cm^3^ (ranging from 4.03 to 32.92 cm^3^). A significant overestimation of tumour volumes obtained using the thresholding techniques could be observed [11, 13, 14], except with the method of Schaefer et al. method [15]. The latter resulted in more accurate estimates compared to the other investigated techniques, but it significantly underestimated tumour volumes as defined on the macroscopic specimens.

The EM delineation overestimated the tumour volume in relation to the reference by a mean volume of 22.4cm^3^. The level set, FCM and FCM-SW methods achieved good results compared to the thresholding and stochastic models; nevertheless, they also tended to slightly underestimate tumour volumes on average. The FCM-SW approach achieved the closest standard deviation to the reference volumes.

**Fig. 5:**
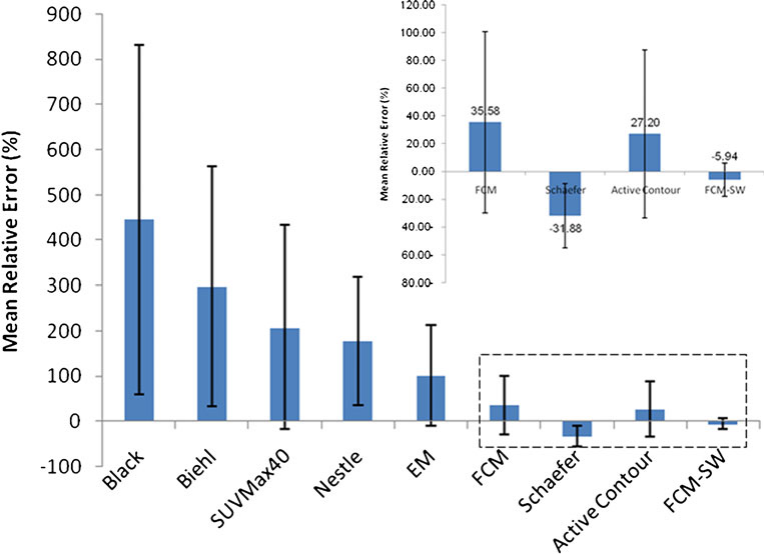
Mean relative volume errors resulting from the different PET image segmentation techniques for the seven clinical cases studied. *Error bars* indicate SDs of the means

Figure 5 shows the mean relative error (calculated as the difference between the measured and the reference volume divided by the reference volume) of the volume estimate for each segmentation technique showing lower errors for the method of Schaefer et al. (−31.9%), the level set method (27.2%), and the FCM-SW method (−5.9%). The mean SOI resulting from the analysis of different PET image segmentation methods relative to the reference defined on the surgical specimens are summarized in Table 1. The levels of statistical significance for paired samples are also shown. The mean SOI varied between 0.27 and 0.54 for the different image segmentation techniques. Again, the FCM-SW segmentation technique showed the best compromise in terms of 3-D SOI and statistical analysis results with values of 0.54 (0.26–0.72) for the SOI.

**Table 1:** Mean SOIs resulting from the different PET image segmentation methods relative to the reference 3-D contour defined on the surgical specimens. The levels of statistical significance for paired samples are also shown

Segmentation method	SOI in relation to histology			*p* value
	Mean	Range	SD	
Black et al. [13]	0.27	0.08–0.68	0.20	<0.005
Biehl et al. [11]	0.33	0.12–0.72	0.20	<0.005
SUV_max40_	0.40	0.15–0.70	0.20	<0.05
Nestle et al. [14]	0.39	0.17–0.70	0.17	<0.002
EM	0.44	0.24–0.62	0.14	0.071
FCM	0.50	0.35–0.63	0.08	0.830
Schaefer et al. [15]	0.43	0.33–0.52	0.07	0.062
Active contour	0.50	0.38–0.63	0.08	0.922
FCM-SW	0.54	0.26–0.72	0.14	0.480

**Fig. 6:**
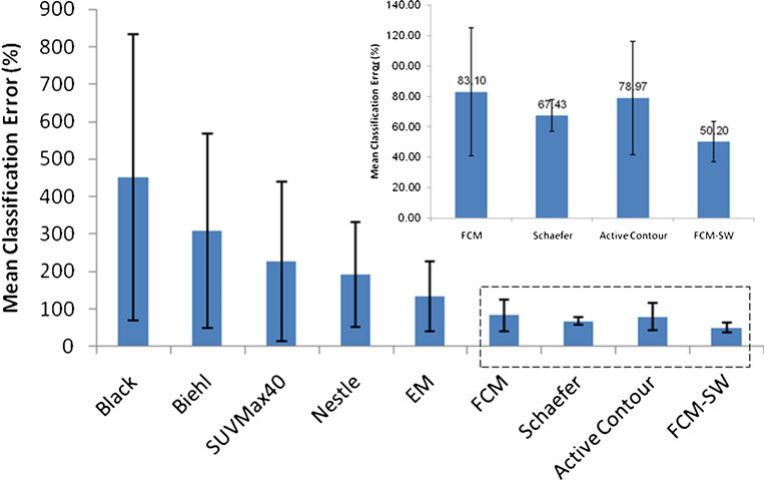
Mean relative CEs computed voxel-by-voxel for the different PET image segmentation techniques. *Error bars* indicate SDs of the means

Figure 6 shows the mean CE achieved by the different PET image segmentation techniques. The CE is a robust measurement of the performance of segmentation algorithms for BTV determination, in contrast to simple volume comparison which can introduce a bias in terms of the location of the segmented lesion. In general, the CE follows a similar trend to the mean relative error. The largest errors were observed for the thresholding techniques (Black et al., Biehl et al., 40% of SUV_max_ and Nestle et al.) and the stochastic EM model. Overall, these algorithms proved to have poor performance for segmenting clinical PET volumes with low contrast resulting in large errors. On the other hand, the method of Schaefer et al., the level set method, and the FCM and FCM-SW methods resulted in substantially lower mean classification errors which varied between 50% (for FCM-SW) and 83% (for FCM) while the EM segmentation approach exhibited a CE of 133.7%.

## Discussion

Molecular imaging-guided clinical diagnosis and radiation therapy treatment planning for pharyngolaryngeal squamous cell carcinoma is highly complex, not only because of the diversity of tumour subsites, but also because of the anatomical restrictions of the head and neck region and the critical importance of preserving the function of organs at risk. The additional information provided by PET/CT combined with sophisticated quantitative methodologies could certainly help to improve diagnostic and treatment strategies. The accurate determination of tumour shape and volume from FDG PET images remains a challenging task owing to the limitations of the current generation of PET scanners, particularly their limited spatial resolution and high noise characteristics resulting from their low sensitivity. Despite the very worthwhile research and development efforts and advances in PET image segmentation [5], there is still scope for improvement given the complexity of the image generation process and the limitations of available techniques, particularly in those with inhomogeneous tumours [31, 32].

This study involved a qualitative and quantitative evaluation of nine PET-based delineation techniques using clinical studies where the 3-D contour defined on in vivo macroscopic surgical specimens served as the reference [7]. Although no reference is perfect, such histological specimens provide the best possible approximation of the tumour boundary. The objective was to select the best available segmentation tool for PET-guided radiotherapy treatment planning and assessment of response to treatment. Although the Louvain database is well documented, the histopathology data were delineated on macroscopic visualization by a single expert histopathologist rather than at the microscopic level and by agreement of an expert panel. In addition, non-uniform uptake of FDG within the tumour volume was lost when compared to the histopathology boundaries, thus excluding the possibility of distinguishing regions in tumours with heterogeneous uptake. Moreover, the data were acquired on an old stand-alone ECAT EXACT HR scanner and reconstructed using predefined parameters, the raw data not being available for reprocessing. These are some of the limitations of this unique dataset. However, it was the only available clinical dataset for pharyngolaryngeal squamous cell carcinoma that provided surgical specimens as reference. We therefore adopted this database as the only available choice for comparison and validation of the investigated PET segmentation techniques. Yet this scanner has a spatial resolution (transaxial spatial resolution varying from 3.6 mm FWHM at the centre to 4.5 mm FWHM tangentially at 20 cm) [33] that is comparable to the resolution achieved by the current generation of PET/CT scanners used to derive actual calibration parameters required by adaptive thresholding approaches [11, 13–15].

The performance of the nine segmentation techniques was highly dependent on the contrast and noise characteristics of the PET images. As such, the histology-derived contours correlated very differently with the PET images shown in Figs. 2 and 3. The PET images used for the comparative assessment were preprocessed following the work of Geets et al. to account for noise and blurring artefacts that may degrade the definition of tumour boundaries [27]. These methods were applied here for consistency with previous work on these data. However, such preprocessing needs to be carefully applied depending on the scanning and acquisition protocols used. All methods used in this work were implemented as described by the authors in their original articles. It is, however, recognized that adaptive thresholding techniques need to be calibrated optimized in the clinic for each PET scanner and acquisition/processing protocol. It should be emphasized that calibration is mandatory for any PET segmentation algorithm to adjust the parameters of the method with respect to the spatial resolution and noise properties of the PET scanner.

The results obtained in clinical studies for various lesion sizes and contrasts demonstrate that none of the methods is adequate for all conditions independent of lesion characteristics and scanning conditions. In this clinically realistic set-up with heterogeneous target and background, thresholding methods [11, 13, 14] performed less well than the method of Schaefer et al. [15]. Moreover, the latter technique, which incorporates prior knowledge of both contrast and volume, performed as well as the more sophisticated approaches assessed in this work. However, the implementation of techniques such as that of Schaefer et al. depends on the scanner, data acquisition and processing protocol, and often requires extensive phantom studies for calibration, which renders their standardization difficult. This makes studies comparing different sites or multicentre clinical trials difficult to carry out. Furthermore, the thresholding methods do not consider inhomogeneities in the BTV [24, 30].

The clinical assessment of segmentation techniques using surgical specimens as reference showed quite similar results to those of previous physical phantom studies [5]. Previous studies in non-small-cell lung cancer have provided similar conclusions, but with small differences owing to the presence of respiratory motion artefacts in thoracic clinical images [34].

The level set and FCM algorithms were a good compromise in terms of the relative error in the volume estimate in comparison with the better performance of the FCM-SW algorithm, with SOI values of up to 0.72 and CEs which were smaller than those with the thresholding, variational and stochastic methods. The CE gives an idea of the spatial location and geometrical shape of the segmented image compared to the reference, and could have values higher than 100%. For this criterion, it was observed that the FCM-SW algorithm out-performed three of the other automated techniques compared in this work, and the method handled typical low-contrast and highly noisy images without clearly defined edges better. The methods of Biehl et al. and Black et al. and the 40% of SUV_max_ algorithm were not successful and gave estimated volumes that were significantly different. In addition, they resulted in higher CEs than all the algorithms evaluated in this work. Overall, the automated methods produced more accurate and robust performance than the thresholding methods except that of Schaefer et al. [15]. The reasons of this exception are not yet clear and are still being investigated using simulated data. Nevertheless, there are still opportunities to refine the algorithms in our study using a larger sample for learning. Another promising direction is combining complementary information from PET and CT images in a joint segmentation process [16].

Geets et al. [27] developed a gradient-based method using the watershed transform and hierarchical cluster analysis and compared it with an adaptive thresholding method based on the signal-to-background ratio approach as implemented by Daisne et al. [35]. Using the same clinical data, the adaptive thresholding method overestimated the actual volume determined in the macroscopic specimens by 68%, whereas the gradient-based approach overestimated the actual volume by about 20%. The FCM-SW approach [24] out-performed both techniques and underestimated the actual volume by only 6% on average.

The results of the study are not straightforward to project to general clinical practice, for example, in the context of the image reconstruction method or acquisition parameters. These parameters are likely to affect the image properties, such as spatial resolution and noise levels, essential for algorithm performance. A more comprehensive assessment of the impact of acquisition, reconstruction and processing techniques on algorithm performance in a more methodical manner over a wider range of resolution/noise levels is required.

### Conclusion

With the limited sample size used in this comparative study, the FCM-SW algorithm achieved the highest accuracy for clinical studies in terms of relative error and overlap in comparison with all the other techniques evaluated for biological target definition in pharyngolaryngeal squamous cell carcinoma. Moreover, it was less parametric than stochastic and variational methods, which could benefit from parameter optimization in some cases. Overall, the BTVs delineated using the FCM-SW technique were the most accurate and approximated closely the 3-D BTVs defined in the corresponding surgical specimens. With slight fine tuning, this technique could be a good candidate for PET-guided radiation treatment planning and assessment of response to treatment. Adaptive thresholding techniques need to be calibrated for each PET scanner and acquisition/processing protocol, and should not be used without optimization.
